# Gpr48 Deficiency Induces Polycystic Kidney Lesions and Renal Fibrosis in Mice by Activating Wnt Signal Pathway

**DOI:** 10.1371/journal.pone.0089835

**Published:** 2014-03-03

**Authors:** Yongyan Dang, Bei Liu, Peng Xu, Pingya Zhu, Yimiao Zhai, Mingyao Liu, Xiyun Ye

**Affiliations:** Shanghai Key Laboratory of Regulatory Biology, Institute of Biomedical Sciences and School of Life Sciences, East China Normal University, Shanghai, China; Ohio State University Comprehensive Cancer Center, United States of America

## Abstract

G protein-coupled receptor 48 (Gpr48/Lgr4) is essential to regulate the development of multiple tissues in mice. The notion that Gpr48 functions in renal development prompted us to investigate the relation between Gpr48 and renal diseases. Using a Gpr48 knockout mice model, we observed that 66.7% Gpr48 null mice developed polycystic lesions in the kidney, while no cysts were observed in the kidneys of wild-type mice. Polycystic kidney disease 1 (PKD1) and PKD2 expressions were also markedly decreased in the Gpr48 knockout mice. Abnormal expressions of exra-cellular matrix protein lead to the progression of polycystic kidney disease and the formation of renal fibrosis in the Gpr48 null mice. The expressions of several Wnt molecules and its receptors were increased and marked β-catenin nuclear accumulation was observed in the Gpr48 null mice. The inhibitors of Wnt/β-catenin signal pathway such as GSK3β and axin2 were loss of function. The Wnt/PCP signaling pathway is also activated in Gpr48 null mice. However, TGF-β expression and phosphorylated Smad2/3 levels were not altered. Collectively, our results showed that Gpr48 null mice are at a greater risk of suffering from polycystic lesions and renal fibrosis. Moreover, the formation of polycystic lesions and renal fibrosis induced by Gpr48 deficiency involves the activation of Wnt signaling pathway but not the TGF-β/Smad pathway.

## Introduction

Human kidneys, responsible for separating urea, mineral salts, toxins and other waste products from the blood, maintain normal life by functioning properly. Kidney diseases can lead to kidney impairment, progressive loss of renal function and end-stage renal failure, which are fatal unless a dialysis machine is used or a kidney transplant is performed. Polycystic kidney disease (PKD) is a renal disease characterized by the growth of numerous cysts filled with fluid in the kidneys. The appearance of interstitial fibrosis in polycystic kidneys is emblematic of progressive disease. Currently, there are no effective drugs for the treatment of PDK and renal fibrosis. Yet, the mechanisms that promote PDK and interstitial fibrosis remain largely unknown.

G protein-coupled receptors (GPCRs) are a large family of transmembrane proteins that are involved in a wide variety of physiological processes. GPCRs can recognize their ligands and transduce extracellular stimuli into intracellular signals by ligand-receptor interactions. Leucine- rich repeat-containing GPCRs (LGRs), a subgroup of the GPCR superfamily, are characterized by the presence of an extracellular domain containing multiple leucine-rich repeats. Gpr48, also called LGR4, belong to type B LGRs with 17 LRRs (leucine-rich repeats) in their putative hormone binding domains. Gpr48 has been demonstrated to be widely expressed in multiple tissues of human and mouse [1]. In addition, Gpr48-deficient mice showed marked intrauterine growth retardation coupled with embryonic and perinatal lethality [2]. The development of several organs, including eye, reproductive gland, hair, bone and gall bladder, is impaired severely when Gpr48 is deficient in the mice [3]–[7]. Also, Gpr48 is demonstrated to be essential for embryonic kidney development in the mice [8].

Although the function of Gpr48 on the development of multiple tissues is apparent, the involvement of Gpr48 in human diseases is less clear. A recent study demonstrated that up-regulation of Gpr48 promoted carcinoma cell invasion and metastasis [9]. GPCR family is also been presumed to be one of the most promising and attractive therapeutic targets for several human diseases such as osteoporosis and neurodegenerative diseases [10]. Since Gpr48 is crucial for maintaining normal renal development and physiological functions [8], we propose that it might also be involved in the formation and development of diseases in the kidney.

In this study, we attempt to elucidate the function of Gpr48 in polycystic kidney lesions and subsequent renal fibrosis as well as the underlying molecular mechanisms. Specifically, we used the *Gpr48^−/−^* and *Gpr48^+/+^* mice as animal models to observe whether Gpr48 deficiency destructs renal structure and function in adult mice. We show that Gpr48 null mice are at a greater risk of polycystic lesions and renal fibrosis. We also provide evidence that the Wnt signal pathway, which is widely known to regulate renal development and diseases [11], [12], is aberrantly activated in the kidney of *Gpr48^−/−^* mice.

## Materials and Methods

### Generation of *Gpr48^−/−^* mutants and genotyping

Gpr48 knockout mice were generated based on the secretory-trap approach as previously described [13] by disrupting the endogenous Gpr48 gene. Genotype of mice was determined by PCR using four primers: for wild type, forward (5′TGT TTC AAC CTT TTA AAG ACT GTA GC3′), reverse (5′TAA AGGACT TAA TGC CAA ATG TGAT3′); for mutant, forward (5′CCA ATC ACC ACT CTT ACA CAA TGG CT 3′), reverse (5′GGT CTT TGA GCA CCA GAG GAC 3′). Genomic DNA was extracted from the clipped tail of 2-week old mice according to the instruction of kit (Tiangen Biotech Co., Beijing, China). PCR was then carried out for 35 cycles at 94°C for 40 s, 53°C for 40 s, and 72°C for 1 min. Products were 450 bp for wild-type and 750 bp for mutant alleles. Thirty mice including ten pairs of littermates (Gpr48+/+ and Gpr48−/−) were used in the study. All mice were kept in the specified pathogen-free (SPF) breeding section, and this study was approved by the Animal Ethical Committee of East China Normal University.

### Biochemical analysis

Blood in the Gpr48 wildtype (WT) and knockout (KO) mice was collected through the retro-orbital venous plexus. Urea nitrogen in sera was determined by using the specific kit (Institute of Nanjing Jiancheng Bioengineering, Nanjing, China) according to the manufacture's protocol.

### Histology and Immunohistochemistry

For histological analysis, kidney tissues were fixed in 4% formaldehyde for 48 hours. 4-µm-thick sections were stained with hematoxylin and eosin. Immunohistochemical analysis was performed by using the ABC staining system (Santa Cruz Biotechnology). The following antibodies from Santa Cruz Biotechnology were used: rabbit polyclonal anti-collagen I (1∶100), rabbit polyclonal anti-MMP2 (1∶100), rabbit polyclonal anti-collagen IV (1∶100), rabbit polyclonal anti-fibronection (FN) (1∶100). Nuclei were counterstained with hematoxylin.

### RT-PCR

The mRNA levels of collagen I, III, IV, MMP, 1, 2, 9, TIMP1, 2, TGF-beta, connective tissue growth factor (CTGF), fibronectin (FN), alpha-smooth muscle actin (α-SMA), intercellular adhesion molecule (ICAM-1), E-cadherin, bcl-2, CD44, TGF-β, PKD1, PKD2, β-catenin, Wnt moleculars and its receptors were measured. Total RNA was isolated from the kidney tissues by using TRIzol reagents according to the manufacture's instruction (Invitrogen, USA). cDNA was synthesized using the M-MLV Reverse Transcriptase Kit (Promega). Real-time PCR was performed using SYBR Premix Ex Taq (TaKaRa). β-actin was used as an internal control. The conditions of PCR reactions were: 35 cycles: 95°C for 30 s, 50°C for 1 min, and 72°C for 30 s. The specific primer sequences used in the study are provided in Table 1.

**Table 1:** Primers for PCR.

Gene	Primer sequence-forward (5′-3′)	Primer sequence-reverse (5′-3′)
β-actin	GGAGACAACCTGGTCCTCAG	ACCCAGAAGACTGTGGATGG
Col I(α2)	CTTGTGGCTTCTGACTATCT	AGGAAAATGAGGCTGTTA
Col III	GCAGTCCAACGTAGATGAATTGG	GAAGGCCTGGTGGACCAGCTGG
MMP1	TTCTGAAACCCTGAGTGC	AAGCCTGGATGCGATTA
MMP2	CTTTGCAGGAGACAAGTTCTGG	TTAAGGTGGTGCAGGTATCTGG
MMP9	CAGCCCCTGCTCCTGGCTCTCCTG	ACTCGTCGTCGTCGAAATGGGCAT
TIMP1	AGTGGGGTCTGTGAGGT	CAAAAGAGGGAGTGCTG
TIMP2	CCGCAAC AGGCGTTTTGCAA	TCACTTCTCTTGATGCAGGC
FN	AGGCTGGATGATGATGGTGGA	GAGTCTGCGGTTGGTAAAT
α-SMA	TACTGCCGAGCGTGAGAT	GCTTCGTCGTATTCCTGTTT
CTGF	TGTGGAATGGGCATCTCC	TTTCATGATCTCGCCATCG
TGF-β	CGGTGCTCGCTTTGTA	GCCACTCAGGCGTATC
Bcl-2	AGGGGGAAACACCAGAATC	GGTAGCGACGAGAGAAGTCA
CD44	AATTCCGAGGATTCATCCCA	CGCTGCTGACATCGTCATC
VCAM-1	CAAGGGTGACCAGCTCATGA	TGTGGCAGCCACCTGAGATCC
E-cadherin	CCTGTCTTCAAC CCAAGCAC	ATTTTCTGACCCACACCAAA
PKD1	TCTGGATGGGCTTCAGCAA	AGCGGGAAGGCAGTGGAT
PKD2	AGACTTCTCGGTGTATAACGCAAA	AGACTTCTCGGTGTATAACGCAAA
Wnt1	GCCCTAGCTGCCAACAGTAGT	GAAGATGAACGCTGTTTCTCG
Wnt2a	AGAGTGCCAACACCAGTTCC	TACAGGAGCCACTCACACCA
Wnt2b	TTGTGTCAACGCCTACCCAGA	ACCACTCCTGCTGACGAGAT
Wnt3	GGGGCGTATTCAAGTAGVTG	GTAGGGACCTCCCATTGGAT
Wnt4	CGAGGAGTGCCAATACCAGT	GTCACAGCCACACTTCTCCA
Wnt5a	CCCAGTCCGGACTACTGTGT	TTTGACATAGCAGCACCAGTG
Wnt5b	TCTCCGCCTCACAAAAGTCT	CACAGACACTCTCAAGCCCA
Wnt6	TTCGGGGATGAGAAGTCAAG	CGGCACAGACAGTTCTCCTC
Wnt7a	GACAAATACAACGAGGCCGT	GGCTGTCTTATTGCAGGCTC
Wnt7b	ACAGGAGGGTGGGGATAGA	GAAACAGCCCAGGAAACCGT
Wnt8a	CTGACTACTGCAACCGCAAC	TGACAGTGCAACACCACTGA
Wnt8b	CCAGAGTTCCGGGAGGTAG	GAGATGGAGCGGAAGGTGT
Wnt9a	CCCCTGACTATCCTCCCTCT	GATGGCGTAGAGGAAAGCAG
Wnt9b	GGGTGTGTGTGGTGACAATC	TCCAACAGGTACGAACAGCA
Wnt10a	GCGCTCCTGTTCTTCCTACT	ATGCCCTGGATAGCAGAGG
Wnt10b	TCAGTCGGGCTCTAAGCAAT	TGGTGCTGACACTCGTGAAC
Wnt16	CCCTCTTTGGCTATGAGCTG	TACTGGACATCATCCGAGCA
Fzd1	CAAGGTTTACGGGCTCATGT	GTAACAGCCGGACAGGAAAA
Fzd2	TCGCCTACAACCAGACCATC	CATTGGAAGCCGAACTTGT
Fzd3	GGGTTGGAAGCAAAAAGACA	CTCCCTGCTTTGCTTCTTTG
Fzd4	CAACCTGTGTGATTGCCTGT	TGTGTGTGGGCTGAAGTGTT
Fzd5	GGCATCTTCAVVVTGCTCTA	TTCCTCTCCAAGCCACTCTG
Fzd6	GGCTGAAGGTCATTTCCAAG	TGAACAGGCAGAGATGTGGA
Fzd7	GAAGCTGGAGAAGCTGATGG	ATCTCTCGCCCCAAATCTCT
Fzd8	CGGTGGTCTTTCTCCTTGTC	TAGAAAAGGCAGGCGACAAC
Fzd9	AGAGCCTGTGCTACCGAAAA	CCCCCTGTGTCTCACTTGTC
Fzd10	GACACCTGACTGCCTGATGA	ACAACCAGCCAACCAAGAAAA

### Preparation of cytoplasmic and nuclear fractions

Kidneys were taken after the mice were killed by cervical dislocation. Then tissues were washed by cold PBS and minced with scissors. After digestion with trypsin for overnight at 4°C and then 30 min at 37°C, tissue samples were vortexed and centrifuged. The pellets were resuspended with PRIM 1640 medium. After deposition for 5 min, the kidney cells in the supernatant were transferred to the 6-cm dishes for the culture.

Primary kidney cells were scraped with cold PBS and resuspended in hypotonic buffer (10 mM HEPES [pH 8.0], 10 mM KCl, 1.5 mM MgCl2, 1 mM DTT) containing protease inhibitor cocktail (Roche) on ice for 15 m. The cells were then Dounce-homogenized and centrifuged at 3000× g for 10 min at 4°C, and the final supernatant (cytoplasmic fraction) was stored at −80°C until use. For preparation of the nuclear extracts, the pellet were wash by cold PBS, isolated by centrifugation and resuspended in nuclear extraction buffer (20 mM HEPES [pH 8.0], 420 mM NaCl, 1.5 mM MgCl2, 0.2 mM EDTA, 25% glycerol, 1 mM DTT, 0.5 mM PMSF, protease inhibitor cocktail) for 40 min at 4°C with brief vortex every 10 min. After centrifugation at 15,000× g for 30 min at 4°C, the supernatant (nuclear fraction) was stored at −80°C. Western analyses were performed using standard protocols as follows.

### Western blot

Kidney tissues were crushed in liquid nitrogen. Then, tissue samples or cell pellets were lysed in NP 40 lysis buffer (50 mM Tris-Cl, pH 8.0. 100 mM NaCl, 5 mM MgCl 2, 0.5% (v/v) Nonidet P-40) on ice for 30 min. The protein concentration was determined using a BCA Protein Assay Kit (Bio-Rad, CA, USA) following the manufacturer's procedure. Samples were run on a 10% SDS-polyacrylamide gel, transferred to an nitrocellulose membrane (Millipore) and immunoblotted with anti-collagen I, IV, anti MMP2, anti-a-SMA, anti-FN, anti-β-catenin, anti-axin2, anti-GSK-3β, anti-akt, anti-JNK, anti-c-jun, anti-c-fos and anti-P-Smad2,3 (Santa Cruz Biotechnology, CA) at 1∶1000 and anti-actin (Santa Cruz Biotechnology, CA) at 1∶5000. Rabbit anti-PA200 serum was diluted 1∶500 into phosphate-buffered saline–0.05% Tween (PBST)–1% ovalbumin. Antigen-antibody complexes were visualized with fluorescent labeled secondary antibodies (Sigma-Aldrich) diluted 1∶5,000 into PBST–1% ovalbumin and Fluorescent Western Blot Imaging Systems (Amersham).

### Statistical Analysis

Statistical analyses were performed with the SPSS 15.0 software. Differences among different groups were evaluated using the student's t-test and p<0.05 was considered to be statistically significant.

## Results

### Gpr48 deficiency causes renal polycystic lesions in mice

**Figure 1:**
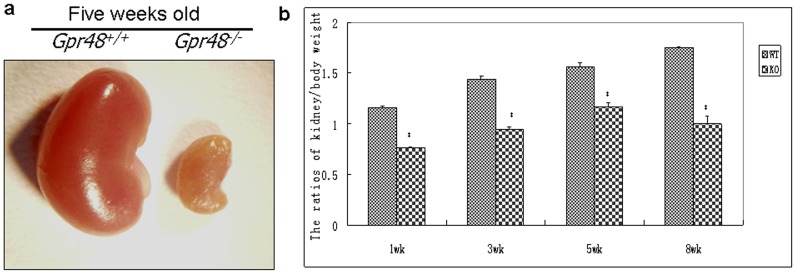
The renal weights and sizes were markedly reduced in the Gpr48 KO mice. (a) The morphology of kidney in five-week old wild-type and knockout mice. Marked polycystic kidney lesions were observed with the naked eyes in the Gpr48 KO mice. (b) The ratios of kidney/body weight in wild-type and knockout mice at different ages.

The average size of kidneys of *Gpr48^−/−^* mice is markedly reduced comparing to wild-type kidneys (Fig. 1a). Significant decreases of renal weight were observed in the Gpr48^−/−^ mice when compared with their wild type littermates. Moreover, the reduction of renal weight in the Gpr48^−/−^ mice was evident at different ages (Fig. 1b). These findings are consistent with previous report that Gpr48 is involving in renal development [8]. Blood urea nitrogen is an indication of renal health. If glomerular filtration rate decreases, urea nitrogen in the serum will increase. In this study, biochemical analysis showed that blood urea nitrogen in Gpr48 KO mice were increased by 38.3% compared with the wild type mice (p<0.05)(Table 2).

**Table 2:** The content of blood urea nitrogen in *Gpr48^+/+^and Gpr48^−/−^* mice.

Mouse type	*Gpr48^+/+^*	*Gpr48^−/−^*
**Blood urea nitrogen (mmol/L)**	7.99±0.81	11.05±1.43*

All data are expressed as Mean ± SD, n = 15.

*p<0.05, ***Gpr48^+/+^*** mice vs ***Gpr48^−/−^*** mice.

**Figure 2:**
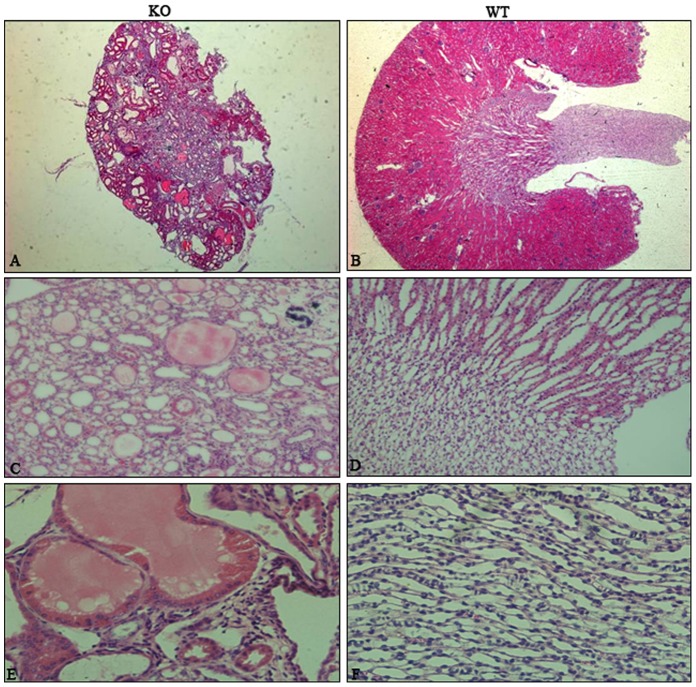
Dominant multiple cysts occurred in the kidneys of the Gpr48 KO mice. The representative morphology of hematoxylin-eosin stains on kidney sections from Gpr48 WT and KO littermates at the adult age. Dominant multiple cysts occurred in the kidneys of the Gpr48 KO mice (A,C,E), whereas the kidneys in the WT mice (B,D,F) were normal. (Magnification, 25× for (A,B), 200× for (C,D), and 400× for (E,F)).

To further understand the function of Gpr48 in renal morphology and function, H&E staining was performed. Polycysts in the kidney were observed in *Gpr48^−/−^* mice [Fig. 2]. For the mice with severe renal impairment, multiple cysts could be seen by naked eyes (Fig. 1a). The renal medulla was severely damaged in the Gpr48 KO mice (Fig. 2). The number of normal nephron was reduced by the expansion of cysts in the *Gpr48^−/−^* kidney. Interestingly, polycystic lesions only occurred in the *Gpr48^−/−^* mice after the age of five weeks. Ten out of fifteen (66.7%) *Gpr48^−/−^* adult mice developed polycysts in the kidneys (Table 3). No cysts were observed in the kidney of all wild-type mice (Fig. 2). Therefore, Gpr48 null mice are at a risk of developing PKD in their near-adult age.

**Table 3:** The incidence of multiple cysts of the kidneys in *Gpr48^+/+^ and Gpr48^−/−^* mice.

Mouse type	*Gpr48^+/+^*	*Gpr48^−/−^*
**Occurrence ratios of multiple cysts**	0/15(0%)	10/15(66.7%)

### Gpr48 deletion causes renal fibrosis in mice

**Figure 3:**
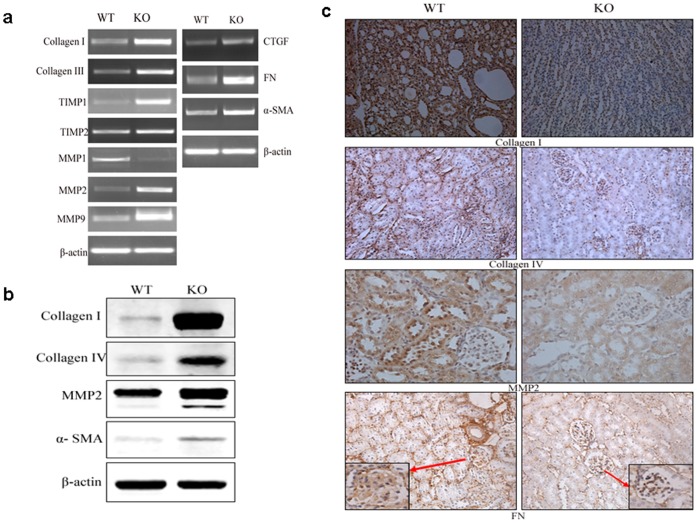
Abnormal expressions of extracellular matrix proteins in the kidneys of Gpr48 KO mice. Some important fibrotic markers are highly expressed in the Gpr48 KO mice compared with the WT mice. (a) Total RNA was extracted and analyzed for the expression of collagenI, III, IV, MMP1,2,9, TIMP1,2 CTGF, FN and alpha-SMA in the kidneys by RT-PCR. (b) Western blot analysis depicted protein level of collagenI, IV, MMP2 and alpha-SMA in the kidneys of mice. (c) The expression of collagen I, IV, MMP2 and FN proteins was studied by immunohistochemistry on Gpr48 KO and WT adult mice (Magnification, 400×).

Renal fibrosis has been reported to associate with the progression of PKD [14]. Therefore, we examined the expression of signaling molecules that are known to be related to renal fibrosis. RT-PCR analysis revealed elevated mRNA levels of collagen I and collagen III in *Gpr48^−/−^* mice (Fig. 3a). The expression of MMP1, an important collagenase, was reduced significantly. In contrast, MMP2 and MMP9 mRNA levels increased markedly. TIMP1 and TIMP2, the inhibitors of MMPs, showed significantly elevated expressions in Gpr48 null mice when compared wild-type littermates (Fig. 3a).

Consistent with mRNA changes, the protein levels of type I and IV collagen were found to increase markedly (Fig. 3b). In addition, immunohistochemical analysis revealed more intense positive staining for type I collagen, type IV collagen and MMP2 in *Gpr48^−/−^* mice than the wild type (Fig. 3c).

The expression of several important fibrotic markers, such as connective tissue growth factor (CTGF), fibronectin (FN) and alpha-smooth muscle actin (α-SMA) were also measured. As expected, the mRNA and protein expression of CTGF, FN and α-SMA increased markedly in *Gpr48^−/−^* mice (Fig. 3). Compared with the wild type, FN showed a nearly 5-fold increase in mRNA level and α-SMA expression was increased by almost 3-fold at both mRNA and protein levels in Gpr48 knockout mice.

### Gpr48 deficiency alters cell adhesion and migration of renal endothelial cells

**Figure 4:**
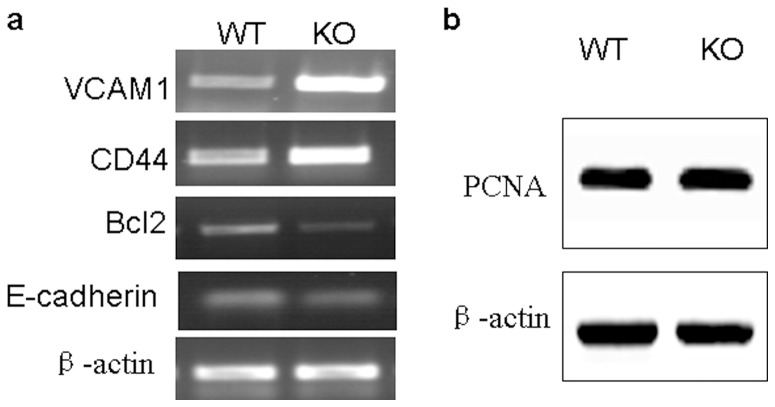
Gpr48 deficiency altered the abilities of cell adhesion. (a) The expressions of ICAM-1, CD44, E-cadherin and Bcl-2 were determined by RT-PCR. The transcript levels of ICAM-1 and CD44 were increased while the expressions of E-cadherin and Bcl-2 were decreased in Gpr48 KO mice compared with the WT mice. (b) Western blot analyses demonstrate PCNA expression is not changed in Gpr48 KO and WT adult mice.

Cellular adhesion molecules, intercellular adhesion molecule (ICAM-1) and CD44, play important roles in renal interstitial inflammation and fibrosis [15]. We found the expression of ICAM-1 and CD44 were enhanced in *Gpr48^−/−^* mice compared with wild-type control (Fig. 4a). Conversely, the mRNA level of E-cadherin was down-regulated in *Gpr48^−/−^* mice (Fig. 4a). B-cell lymphoma 2 (Bcl-2), an important inhibitor of cell apoptosis, was reduced markedly in the kidney of *Gpr48^−/−^* mice (Fig. 4a). Western blot analysis revealed that the expression of cell proliferation marker PCNA was not affected (Fig. 4b).

### Gpr48 deletion leads to elevated expressions of PDK1 and PDK2

**Figure 5:**
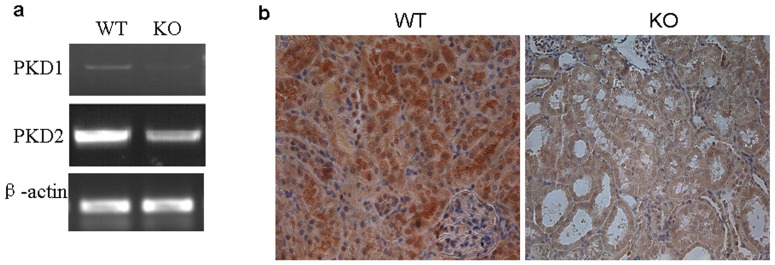
Gpr48 deficiency inhibited the expressions of PKD1 and PKD2. (a) The expressions of PKD1 and PKD2 were determined by RT-PCR. The mRNA levels of PKD1 and PKD2 were significantly decreased in Gpr48 KO mice compared with the WT mice. (b) Representative micrographs demonstrated the protein expression of PC1 was markedly lower in Gpr48 KO mice than in the WT adult mice. Kidneys from Gpr48 KO and WT adult mice were stained immunohistochemically for PC-1 protein. **(Magnification, 400×)**.

Mutations in PDK1 and PDK2 genes have been demonstrated to be responsible for the formation of PKD. Therefore, we examined the expressions of PDK1 and PDK2 by RT-PCR and immunohistochemistry. Indeed, the levels of both PDK1 and PDK2 mRNA were downregulated markedly in Gpr48 KO mice when compared with the wild-type mice (Fig. 5a). Moreover, we observed that polycystin-1, a large transmembrane protein encoded by the *Pkd1* gene, was widely expressed at a high level in the kidney of wild-type mice, whereas its expression was weak in Gpr48 knockout mice (Fig. 5b).

### Gpr48 deletion affects the activity of Wnt signal pathway

**Figure 6:**
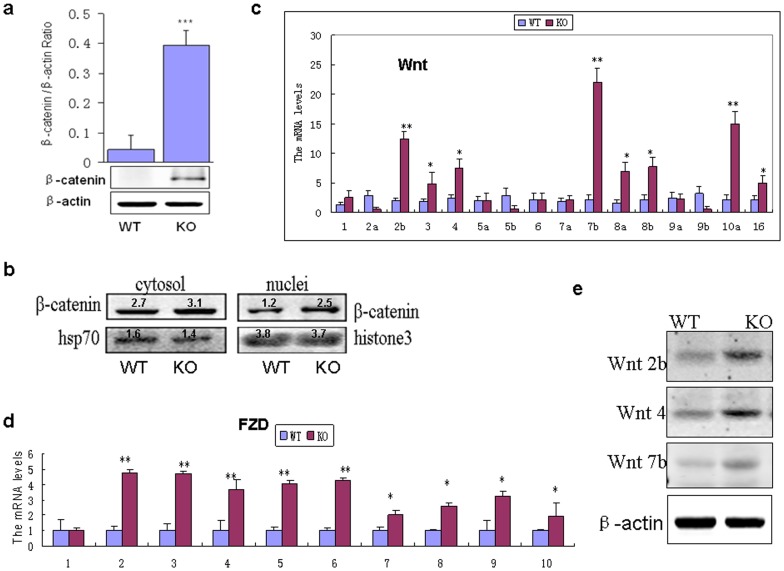
β-catenin/Wnt signaling was activated in the kidneys of *Gpr48^−/−^* mice. (a) The expression of β-catenin was analyzed by Western blot. (b) Western analyses of β-catenin in cytolosic and nuclear fractions in the kidney cells of Gpr48 KO and WT mice. (c)RT-PCR results showed the expressions of different Wnt genes in the kidneys of Gpr48 KO and WT adult mice. (d) The expressions of different Fzd genes were evaluated by RT-PCR in the kidneys of Gpr48 KO and WT adult mice. (e) Western blot analysis shows the dramatic increases of Wnt2b, Wnt4b and Wnt7 in Gpr48 KO mice. All data are expressed as Mean ± SD, n = 15. ***p<0.001, ** p<0.01,*p<0.05, KO vs WT.

Wnt signaling has been reported to be hyperactive and detrimental in the evolution of PKD and renal interstitial fibrosis [16], [17]. This led us to examine the expression of Wnt signaling molecules. In this study, β-catenin protein was accumulated in the kidneys of Gpr48 KO mice but not their wild-type littermates (Fig. 6a). The data of nuclear and cytoplasmic protein fractionation showed that the active form of β-catenin in the nucleus was more in Gpr48 KO than the wild-type mice [Fig. 6b]. Additionally, the mRNA levels of Wnt1, Wnt2b, Wnt3, Wnt4, Wnt7a, Wnt7b, Wnt8a, Wnt8b, Wnt9a, Wnt10a and Wnt16 showed significant increases in the *Gpr48^−/−^* mice ( Fig. 6c). The mRNA expressions of Frizzled (FZD), receptors of Wnt, were also increased (Fig. 6d). In contrast, the expressions of Wnt2b, Wnt5b and Wnt9b were reduced in the *Gpr48^−/−^* mice (Fig. 6c). Furthermore, the protein expression of Wnt2b, Wnt4 and Wnt7b was increased in Gpr48 KO mice (Fig. 6e).

**Figure 7:**
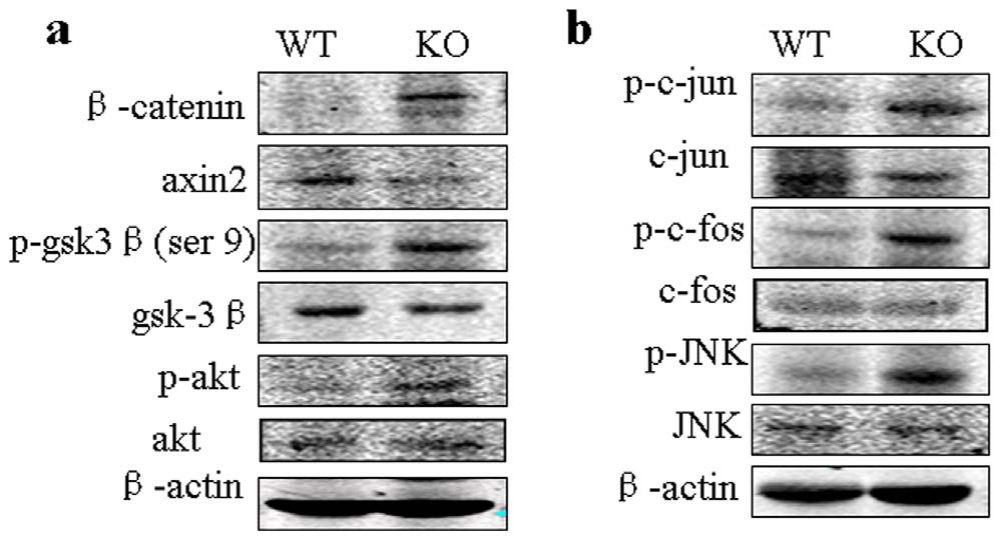
Gpr48 deficiency altered the expression of Wnt signal inhibitors and the activity of noncanical Wnt signal pathway. (**a**) Western blot analysis shows that p-akt and p-GSK3βwere increased while axin2 was decreased in gpr48 KO mice. (**b**) Western blot analysis depicts the levels of p-JNK, p-c-jun and p-c-fos in the kidneys of Gpr48 KO and WT adult mice.

Phosphoinositide 3-kinase/protein kinase B (PI3K/Akt) signaling pathway has been reported to be involved to inhibit GSK-3β kinase in renal cells. Phosphorylation of GSK-3β at the Ser9 residue is known to inactivate the function of GSK-3β kinase [18].We observed that the phosphorylation of akt was significantly enhanced in Gpr48 KO mice compared with the wild-type mice (Fig. 7a). As expected, the level of p-GSK-3β was increased in Gpr48 KO mice (Fig. 7a). Axin2, the other inhibitory factor of Wnt signal pathway, was markedly decreased in the Gpr48 KO mice (Fig. 7a).

It is known that JNK pathway is involved in the noncanonical pathway (the Wnt/PCP signaling pathway). To confirm if Wnt/PCP signaling pathway is involved in the formation of polycystic lesions in Gpr48 null mice, we detected the activity of JNK and its downstream AP1. As showed in Figure 7b, Gpr48 deletion caused the marked upregulation of the levels of p-JNK, p-c-fos and p-c-jun.

### Gpr48 deletion does not alter the activity of the Smad pathway

**Figure 8:**
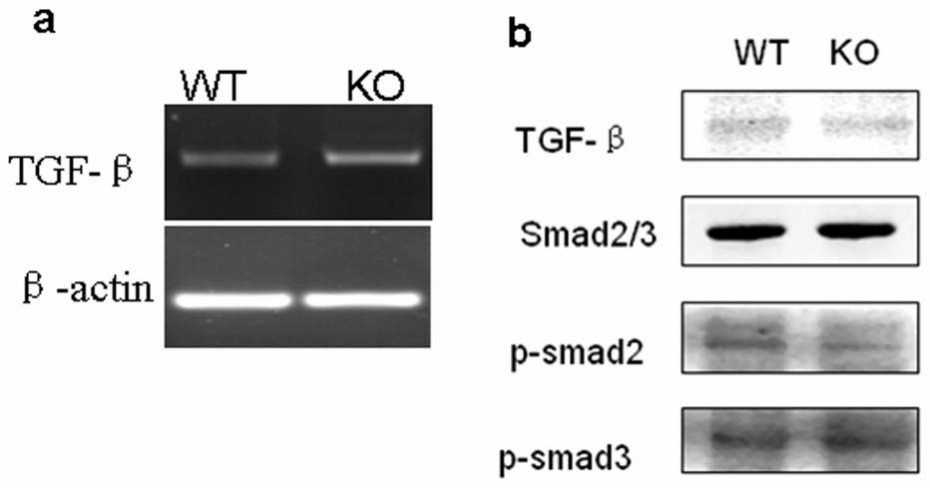
TGF-β1/Smad signaling was not altered in the kidneys of Gpr48 KO mice. (a) TGF-β1 expression in the kidney of mice was evaluated by RT-PCR. (b) The phosphorylated forms of smad2 and smad3 were analyzed by western blot.

TGF-β signal pathway is an important contributor to extracellular matrix protein accumulation and renal fibrosis. However, no marked difference in TGF-β mRNA expression was observed between *Gpr48^+/+^* and *Gpr48^−/−^* mice (Fig. 8a). Moreover, both the total Smad2/3 and phosphorylated Smad 2/3 showed similar levels between *Gpr48^+/+^* and *Gpr48^−/−^* mice (Fig. 8b).

## Discussion

Gpr48 has been demonstrated to be highly expressed in the kidneys of mice when compared with the other organs such as heart, lung and liver [11]. Moreover, striking renal hypoplasia and glomerulus decrease were found in the LGR4 null mice, which suggest that Gpr48 was essential for renal development in mice [11]. Thus, we conjectured that Gpr48 gene may be one of a group of genes responsible for kidney diseases. Due to the high mortality rate of Gpr48 null mice, we generated Gpr48 knockout mice that were spared the embryonic/neonatal lethality by abundant breeding and crossing. We observed severely reduced kidney in size and weight from newborn to adult, which is similar with the previous work [11]. The kidney function was also abnormal in Gpr48 knockout mice compared with the wild type mice. Furthermore, about 66.7 percent of Gpr48 knockout mice at their adult age developed dominant polycysts in the kidney, while no cysts were observed in wild type mice at the same age. Our results indicated that Gpr48 knockout mice are at greater risk of suffering from polycystic kidney lesions than the wild type mice. Simultaneously, we observed β-catenin and Wnt signal molecules were up-regulated in the Gpr48 null mice. Thus, we assumed that polycystic kidney lesions induced by deletion of Gpr48 was related with Wnt signal pathway.

The basement-membrane structure, interstitial matrix composition and the expression of matrix metalloproteases were demonstrated to be abnormal in patients with polycystic kidney diseases. Since the progression of polycystic kidney is actually the course of renal fibrosis [19], we further measured the expressions of extracellular matrix proteins and several important fibrotic markers in the kidney of mice. As expected, collagen Iα2, III, IV, FN and α-SMA were markedly up-regulated in the Gpr48 null mice, indicating the overdeposition of the extracellular matrix proteins and the increase of basement-membrane thickness. In addition, the down-regulation of matrix collagenase MMP1 and the elevation of TIMP1and TIMP2 in the Gpr48−/− mice suggested the inhibition to the degradation of matrix proteins. These results showed both increasing matrix synthesis and decreasing degradation contributed to the abnormal accumulation of interstitial matrix in the kidney of *Gpr48^−/−^* mice. Surprisingly, MMP2 and MMP9 expression in the *Gpr48^−/−^* mice were upregulated when compared with the wild type mice. A study reported that MMP2 and MMP9 are potentially capable of disrupting the kidney architecture by virtue of their specificity for various components of basement membrane, thus enabling further infiltration of inflammatory cells into the kidney and thereby setting into motion of the dysregulated matrix remodeling that follows [20]. Thus, we speculated that MMP2 and MMP9 play a role in the formation of polycystic kidney lesions and renal fibrosis in a different way from MMP1.

Wnt/β-catenin signaling has been shown to play a role in kidney development and diseases [21], [22]. Many Wnt signaling components have been implicated in kidney development including Wnt-1, Wnt-2b, Wnt-4, Wnt-6, Wnt-7b, Wnt-9b, Wnt-11 [23]–[25]. At the presence of Wnt, β-catenin is permitted to accumulate in the cytosol and can be subsequently translocated into the nucleus to perform a variety of functions. However, Wnt signaling is silenced in the adult kidney and reactivated after renal injury [21]. Interestingly, ectopic activation of Wnt signaling cascade is associated with polycystic kidney lesions [26]. In this study, we found the expressions of several Wnt molecules including Wnt-2b, Wnt-4, Wnt-7, Wnt-8 and Wnt-10, Frizzle receptors and nuclear β-catenin were markedly upregulated in the Gpr48 null mice compared with the wild-type littermates. The inhibitors of Wnt signal pathway such as axin2 and GSK-3β showed partly loss of function. All the data suggested that Wnt/β-catenin signaling pathway were activated in the Gpr48 null mice. Since MMP2 and MMP9 are known to be the targets of β-catenin-dependent Wnt signaling, the marked alteration of MMP2 and MMP9 expression may be a functional consequence of altered Wnt signaling. Recent studies demonstrated that Wnt4 was involved in the pathogenesis of renal fibrosis [27], which is consistent with our results. However, the expression of Wnt2a, Wnt5b, and Wnt9b were found to be slightly reduced in our study. A similar phenomenon was described in a previous work, in which Wnt4 increased greatly in the fibrotic kidney while Wnt9b were unaltered [22]. We speculated that Wnt2a, Wnt5b and Wnt9b might not participate in the formation of polycystic kidney lesions and renal fibrosis in the Gpr48 null mice.

Interestingly, we observed that p-Akt was increased in Gpr48 KO mice, which can phosphorylate its downstream target GSK-3β. As expected, the levels of p-GSK-3β (ser 9) in the Gpr48 knockout mice were markedly increased. Obviously, GSK-3β activity was inhibited in Gpr48 null mice because phosphorylation at Ser9 is the inactivated form of GSK-3β. Loss of function of Wnt signal inhibitors such as axin2 and GSK-3β may further promote the activation of Wnt signal pathway induced by deficiency of Gpr48. Because axin2 is a direct transcriptional target of Wnt signaling, it could act in a negative feedback loop to limit Wnt signaling. Some studies reported that axin2 could be induced by canonical Wnt signaling [28]. However, we observed that the protein level of axin2 in Gpr48 KO mice was lower while the level of β-catenin was higher. A previous study reported that the binding of axin to the Wnt coreceptor LRP as well as to Dvl could result in the dephosphorylation of axin, thus leading to a decrease in its affinity for β-catenin, and in a decrease in the level of axin [29]. We assumed that the decrease of axin2 protein in Gpr48 KO mice may be related with its degradation due to the persistent activation of Wnt signal pathway, which needs more researches in the future.

The noncanonical pathway includes 3 pathways (the Wnt/Ca^2+^, Wnt/G protein, and Wnt/PCP signaling pathways). These pathways primarily regulate cell movement. Among these, it is believed that the JNK pathway is involved in the noncanonical pathway (the Wnt/PCP signaling pathway) [30]. In this study, we observed that the levles of p-JNK, p-c-jun and p-c-fos were increased in Gpr48 null mice, indicating that Wnt/PCP pathway is also important in cystogenesis in the Gpr48 null mice. Together, we surmise that the activation of both canonical and noncanonical Wnt signal pathways contribute to the formation of polycystic kidney lesions and the subsequent renal fibrosis in the Gpr48 null mice.

It is reported that ADPKD is caused by an inherited mutation in either PKD1 or PKD2 that accounts for 85% and 15% of cases, respectively. In this study, the expression of PKD1 and PKD2 was inhibited in Gpr48 null mice compared with the wild type mice, suggesting that polycystic kidney lesions in Gpr48 KO mice were related with the decrease of PKD expression. PKD1 has a 12.9 kb open reading frame that encodes polycystin-1 (PC1). As for the effect of PC1 on Wnt signaling, the conclusions are contradictory. Some studies showed that the carboxyl (C) terminus of PC1 augments the canonical Wnt transcriptional response [16], whereas other studies reported a potential inhibitory effect [21]. Our data showed that Gpr48 deficiency markedly reduced the expression of PC-1 and activated the canonical Wnt signal pathway. Thus, our results seem to support the idea that PC-1 plays an inhibitory effect on Wnt signal pathway in the mouse kidney. Meanwhile, we observed that, the p-c-jun and p-c-fos were upregulated in Gpr48 null mice. Interestingly, in the promoter of PKD1, there is the binding site of AP1. We thus speculated that AP1 may suppress the expression of PKD1 as an inhibitory transcriptional factor, which need further researches. Consistently, Omori S et al reported that the expression of MAPK is dysregulated in cyst epithelium in pcy mice (a murine model of polycystic kidney disease) [31].

E-cadherin, a member of the cadherin family, can bind β-catenin and prevent its translocation to the nucleus. The E-cadherin-catenin complex plays a crucial role in epithelial cell-cell adhesion and in the maintenance of tissue architecture [32]. In the Gpr48 knockout mice, the decrease of E-cadherin might be partly responsible for the high activity of Wnt signal pathway. Metalloproteases can cleave E-cadherin extracellularly, generating a soluble fragment that impairs cell-cell adhesion [33]. Therefore, the decrease of E-cadherin in Gpr48 knockout mice may be related with the increase of MMP2 and MMP9. Cellular adhesion molecules, ICAM-1 and CD44, play important roles in renal interstitial inflammation and fibrosis. Under normal conditions, CD44 and its ligands are hardly expressed in the kidney, but upon injury, their expression is markedly enhanced. Experiments in animals have shown that targeting of CD44 by antibodies, antisense oligonucleotides, and CD44-soluble proteins markedly reduces the malignant activities of various neoplasms [34]. ICAM-1 expression is reported to be upregulated by CD44 and associated with leukocyte infiltration in human glomerulonephritis [35]. In this study, the altered expression of E-cadherin, Vcam1, and CD44 in Gpr48 knockout mice suggests that the epithelial layer of kidney was not intact in Gpr48 knockout mice. Bcl-2 protein mediates anti-apoptotic signals in a wide variety of human cell systems. The level of bcl-2 in Gpr48 knockout mice was lower than the wild-type mice, suggesting that the renal epithelial cells were damaged and at a higher risk of apoptosis. We surmise that Gpr48 deficiency causes renal polycystic lesions may be also related with altering renal epithelial cell adhesion and apoptosis.

TGF-β is well known to be a potent mediator of renal fibrosis, and Smad proteins are critical intracellular mediators of TGF-β signaling [36]. TGF-β mediates renal fibrogenesis in tubular epithelial cells in association with the activation of Smad2 [37]. The activity of the TGF-β/Smad signaling pathway was demonstrated to be elevated in different *Pkd1* mutant mouse models and in human ADPKD tissues [14]. However, we found little evidence of altered TGF-β expression/signaling in the Gpr48 null mice. Therefore, TGF-β/Smad pathway is unlikely to be involved in the regulation of the renal fibrosis induced by Gpr48 deletion.

In summary, our data suggest that Gpr48 ablation results in polycystic kidney lesions in the kidney of adult mice by decreasing the expression of PKD1 and PKD2. Abnormal accumulation of exracelluar matrix proteins and altered cell adhesion of renal endothelial cells were associated with the progression of polycystic kidney lesions in the Gpr48 null mice. The formation of polycystic kidney lesions and renal fibrosis induced by Gpr48 deficiency involves the activation of canonical and noncanonical Wnt signaling pathways but not TGF-β/Smad pathway. These results suggest Gpr48 may have the potential to be an attractive therapeutic target for polycystic kidney lesions and renal fibrosis in the future.
